# Occlusal Dysesthesia (Phantom Bite Syndrome): A Scoping Review

**DOI:** 10.3390/dj14010047

**Published:** 2026-01-12

**Authors:** Ivica Pelivan, Sven Gojsović, Samir Čimić, Nikša Dulčić

**Affiliations:** 1 Department of Removable Prosthodontics, School of Dental Medicine, University of Zagreb, Gundulićeva ulica 5, HR-10000 Zagreb, Croatia; pelivan@sfzg.unizg.hr (I.P.); scimic@sfzg.unizg.hr (S.Č.); 2 Department of Fixed Prosthodontics, School of Dental Medicine, University of Zagreb, Gundulićeva ulica 5, HR-10000 Zagreb, Croatia; sgojsovic@sfzg.unizg.hr

**Keywords:** occlusal dysesthesia, phantom bite syndrome, occlusal hyperawareness, oral cenestopathy, somatoform disorder

## Abstract

**Background**: Occlusal dysesthesia (OD), also known as phantom bite syndrome, is characterized by the subjective sensation of an uncomfortable or “wrong” bite despite the absence of objective occlusal pathology. This scoping review aimed to synthesize the current evidence on the epidemiology, etiology, clinical presentation, diagnosis, and management of OD. **Methods**: The PubMed, Google Scholar, Scopus, Web of Science, ScienceDirect, and Cochrane Library databases were systematically searched using the terms “phantom bite,” “occlusal dysesthesia,” “occlusal hyperawareness,” “occlusal hypervigilance,” “uncomfortable occlusion,” and “oral cenestopathy.” Studies were screened according to the Preferred Reporting Items for Systematic Reviews and Meta-Analyses criteria (2020), and evidence quality was assessed using the Oxford Center for Evidence-Based Medicine levels of evidence. **Results**: A total of 20 studies were included. OD predominantly affected middle-aged women, with symptom durations often exceeding several years, and was believed to be caused by disorderly central sensory processing or maladaptive signal processing rather than by a primary occlusal abnormality, with high rates of psychiatric comorbidities reported. Current evidence supports conservative multidisciplinary management, including patient education, cognitive behavioral therapy, and supportive pharmacotherapy, and irreversible dental interventions are contraindicated. **Conclusions**: OD is a complex biopsychosocial condition requiring multidisciplinary care. The current low-quality evidence is primarily obtained from case reports and case series. Therefore, high-quality controlled trials are urgently required to establish evidence-based diagnostic criteria and treatment protocols.

## 1. Introduction

Occlusal dysesthesia (OD), also known as phantom bite syndrome (PBS), is a challenging clinical entity characterized by persistent complaints of an uncomfortable, altered, or “wrong” bite sensation in the absence of objectively verifiable occlusal discrepancies [1,2]. This condition poses significant diagnostic and therapeutic challenges for dental practitioners, as affected patients often undergo multiple unsuccessful dental interventions in pursuit of relief, potentially leading to iatrogenic complications and symptom worsening [3,4].

The terminology used to describe this condition has evolved over time, reflecting changing conceptual understanding. Early studies used terms such as “phantom bite” to emphasize the discrepancy between subjective perception and objective findings [5]. In contrast, contemporary literature increasingly favors “OD” to highlight the abnormal sensory perception underlying the condition [1,6]. Related terms in the literature include “occlusal hyperawareness,” “occlusal neurosis,” “persistent uncomfortable occlusion,” and “oral cenestopathy” [2,7,8].

Historically, OD was often classified as a primary psychiatric disorder, with some authors describing it as a monosymptomatic hypochondriac or delusional condition [9]. However, recent evidence allows a more nuanced understanding, and OD is believed to be caused by disorderly central sensory processing or maladaptive signal processing, with contributions from both neurophysiological and psychological factors [1,10,11]. This paradigm shift has implications for clinical management, emphasizing the importance of conservative multidisciplinary approaches over aggressive dental interventions.

Despite growing clinical recognition, OD remains under-researched, with limited epidemiological data and a paucity of high-quality interventional studies [2,6]. The impact of this condition on patients’ quality of life can be substantial, and it is characterized by persistent distress, preoccupation with oral sensations, and multiple visits to various practitioners [9,10].

Considering the complex and often misunderstood nature of OD, comprehensive synthesis of existing evidence is critical to inform clinical practice and identify research gaps. The absence of robust evidence, the inconsistent terminology, and the poorly structured OD literature facilitate the integration of information through a scoping review approach. Therefore, this scoping review aimed to systematically map the available literature on OD; synthesize the current knowledge regarding its epidemiology, etiology, clinical features, diagnostic approaches, and management strategies; and provide evidence-based guidance to clinicians encountering this challenging condition. Additionally, this scoping review aimed to assess the quality of available evidence using the Oxford Center for Evidence-Based Medicine (CEBM) levels of evidence and identify gaps in the current knowledge and priorities for future research.

## 2. Materials and Methods

### 2.1. Protocol and Registration

This scoping review adhered to the Preferred Reporting Items for Systematic Reviews and Meta-Analyses extension for Scoping Reviews (PRISMA-ScR) guidelines [12]. The completed PRISMA-ScR checklist is provided as Supplementary Material File S1. The protocol was prospectively registered with PROSPERO (registration ID CRD420251157506).

### 2.2. Eligibility Criteria

#### 2.2.1. Inclusion Criteria

Studies were included if they met the following criteria:

Population: Studies involving patients who report persistent occlusal discomfort in the absence of objective occlusal pathology. This includes various terminology, such as occlusal dysesthesia, phantom bite syndrome, occlusal hyperawareness, oral cenestopathy, occlusal discomfort syndrome, uncomfortable occlusion, and other related terms.

Study Designs: All study designs were eligible for inclusion, including systematic reviews, meta-analyses, randomized controlled trials, cohort studies (both prospective and retrospective), case–control studies, case series (with two or more cases), case reports, clinical guidelines, consensus statements, cross-sectional surveys, narrative reviews, and theoretical or conceptual articles.

Language: Studies published in English or German, or those with available English abstracts, were included.

Publication Status: Only peer-reviewed journal articles were considered.

#### 2.2.2. Exclusion Criteria

Studies were excluded based on the following criteria:

Off-topic studies: Articles that focus exclusively on general temporomandibular disorders (TMD), orofacial pain, or burning mouth syndrome, without specific mention of occlusal dysesthesia or phantom bite.

Non-human studies: Research involving animals or in vitro experiments.

Purely technical articles: Studies that concentrate solely on occlusal analysis techniques or dental materials, lacking clinical relevance to occlusal dysesthesia or phantom bite syndrome (OD/PBS).

Duplicate publications: Instances of multiple publications presenting the same data, with the most recent or comprehensive version retained.

Inaccessible full texts: Studies for which the full text could not be obtained even after contacting the authors.

Language: Articles published in languages other than English or German.

Publication status: Conference abstracts, dissertations, book chapters, and grey literature, with the exception of clinical guidelines from recognized professional organizations.

### 2.3. Information Sources and Search Strategy

A comprehensive literature search was conducted in October 2025 using the PubMed, Google Scholar, Scopus, Web of Science, ScienceDirect, and Cochrane Library databases to ensure broad coverage of the available evidence.

The following search terms were used individually and in combination using Boolean operators:

(“Phantom bite” [Title/Abstract] OR “Occlusal dysesthesia” [Title/Abstract] OR

“Occlusal hyperawareness” [Title/Abstract] OR “Occlusal hypervigilance” [Title/Abstract] OR 

“Uncomfortable occlusion” [Title/Abstract] OR “Oral cenestopathy” [Title/Abstract])

### 2.4. Selection Process

The selection process followed the PRISMA guidelines (2020) and consisted of multiple stages. Studies were identified via the initial searches across all databases, and duplicates were removed. Before the comprehensive screening, the two reviewers (I.P. and S.G.) independently conducted a pilot screening on a random sample of 20 records to guarantee uniform application of inclusion and exclusion criteria. Discrepancies during the pilot phase were addressed, and criteria were enhanced to augment inter-rater agreement prior to initiating comprehensive screening.

Next, two independent reviewers (I.P. and S.G.) screened the titles and abstracts of the identified studies according to the eligibility criteria. Finally, the full texts of articles that passed the initial screening were reviewed to determine the eligibility for inclusion. Disagreements at any stage were resolved through discussion and consensus of all four reviewers.

### 2.5. Data Collection

For each included study, the following data were systematically extracted: Bibliographic information (authors, year, title, journal, and Digital Object Identifier), study design and type, sample size (number of participants, cases, or studies reviewed), study population characteristics (age, sex, and demographics), clinical presentation and symptom characteristics, etiological hypotheses and proposed mechanisms, diagnostic criteria and assessment methods, treatment approaches and interventions, outcomes and prognosis, and key findings and conclusions.

### 2.6. Quality Assessment

The quality of evidence was assessed using the Oxford CEBM levels of evidence as follows [13]:

Level 1: Systematic reviews, meta-analyses, randomized controlled trials.

Level 2: Cohort studies, low-quality randomized controlled trials.

Level 3: Case–control studies.

Level 4: Case series, case reports, and poor-quality cohort/case–control studies.

Level 5: Expert opinion, mechanism-based reasoning, and clinical guidelines.

### 2.7. Data Synthesis

Given the heterogeneity of design and predominantly descriptive nature of the included studies, a qualitative narrative synthesis approach was used. The findings were organized thematically according to the review objectives, with a chronological presentation of evidence development, where appropriate, to illustrate the evolution of understanding over time.

## 3. Results

### 3.1. Study Selection

**Figure 1:**
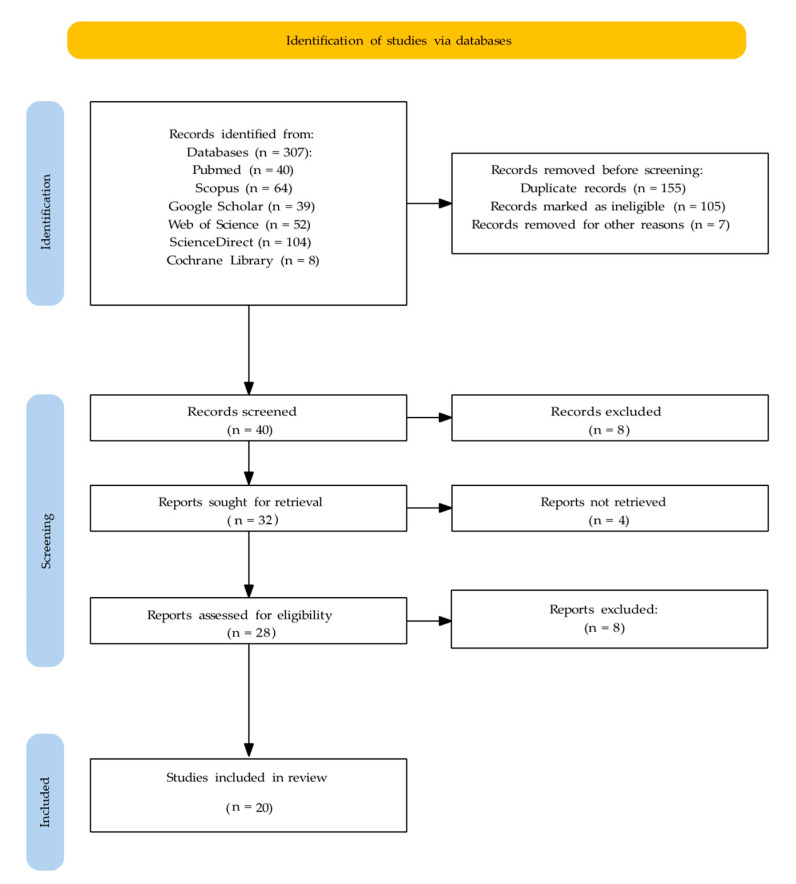
PRISMA flowchart illustrating the study selection process.

The PRISMA 2020 flow diagram (Figure 1) illustrates the study selection process.

The database searches retrieved a total of 307 studies, of which 40, 64, 39, 52, 104 and 8 studies were retrieved from the PubMed, Scopus, Google Scholar, Web of Science (via web search), ScienceDirect (via web search), and Cochrane Library (via web search) databases, respectively.

After merging the database results and removal of duplicates, 40 unique records were screened based on their titles and abstracts. Of these, 8 were excluded (not relevant), and 32 met the criteria for full-text retrieval. Of these, full texts of four reports could not be retrieved; thus, 28 studies were assessed for eligibility. After full-text assessment, eight reports were excluded (wrong focus). Finally, 20 studies were included in the analysis.

### 3.2. Study Characteristics

The 20 studies included one systematic review [2], one theoretical article [14], four narrative reviews [5,6,11,15], two clinical guidelines [1,16], two retrospective case series [7,17], two case–control studies [18,19], one survey [20], one retrospective cohort study [10] and six case reports [3,4,8,21,22,23]. All studies were published between 2007 and 2025, and the majority were published after 2012, reflecting the growing clinical and research interest in this condition.

### 3.3. Evidence Quality Distribution (Oxford CEBM)

The evidence was classified as Level 1, 2, 3, 4, and 5 in 0 (0%), 1 (5%) [2], 2 (10%) [18,19], 4 (20%) [7,10,17,20], and 13 (65%) [1,3,4,5,6,8,11,14,15,16,21,22,23] studies, respectively.

### 3.4. Synthesis of Findings

The results are organized thematically according to the scoping review objectives, addressing:

Section 3.4.1. Terminology and Definitions;

Section 3.4.2. Epidemiology and Demographics;

Section 3.4.3. Clinical Presentation;

Section 3.4.4. Etiology and Pathophysiology;

Section 3.4.5. Differential Diagnosis;

Section 3.4.6. Diagnostic Approaches and Criteria;

Section 3.4.7. Treatment Approaches and Management.

This structure facilitates systematic synthesis of the heterogeneous literature on OD.

#### 3.4.1. Terminology and Definitions

The analysis revealed considerable terminological variation in the literature, reflecting evolving conceptual frameworks. “Phantom bite” was among the earliest descriptive terms used in clinical reports and remains common in dental parlance [5,9]. This term emphasizes the phantom-like quality of symptoms that lack objective correlations. However, OD has emerged as the preferred term in recent clinical guidelines and reviews [1,2,6]. This term emphasizes abnormal perception (dysesthesia) rather than suggesting a primary occlusal defect. Related and alternative terms include “Occlusal hyperawareness” or “occlusal hypervigilance” [7], “persistent uncomfortable occlusion” [7], “occlusal neurosis” (historical term), and “oral cenestopathy” (rare). The term “oral cenestopathy” appears predominantly in Japanese psychiatric literature [11], reflecting the Japanese diagnostic tradition that recognizes cenestopathy (abnormal bodily sensations) as a distinct diagnostic category.

Despite this terminological heterogeneity, all terms describe the same core phenomenon—persistent occlusal discomfort without objective occlusal pathology. Current literature and clinical guidelines [14,15,16] favor “occlusal dysesthesia (OD)” as the primary term, with “phantom bite syndrome (PBS)” as an acceptable and widely recognized synonym. For consistency and clarity, this scoping review uses “occlusal dysesthesia (OD)” as the primary designation, with acknowledgment of “phantom bite syndrome (PBS)” as a synonym.

Across studies, OD was consistently defined as a persistent complaint of uncomfortable, altered, or “wrong” bite sensation despite the absence of objectively verifiable occlusal discrepancy, typically lasting months to years [1,2,6]. However, the concept of OD has evolved significantly over time. Early studies (pre-2010) emphasized psychiatric etiologies, with some authors characterizing OD as a monosymptomatic hypochondriac or delusional disorder [9]. Yamaguchi et al. (2007) described “persistent uncomfortable occlusion” and emphasized the heterogeneous clinical presentations and variable treatment responses [7].

Hara et al. (2012) reported the first systematic review, proposed unified diagnostic criteria, and acknowledged multiple etiologies [2]. Subsequently, biopsychosocial models, such as that by Melis and Zawawi (2015), emphasized the contraindications for irreversible dental treatments [6]. Later, the landmark clinical guidelines by Imhoff et al. (2020) reframed OD as a maladaptive sensory or signal-processing disorder rather than a primary occlusal abnormality and recommended conservative multidisciplinary management [1]. Recent reviews by Tu et al. (2021, 2022) and Türp and Hellmann (2023) reinforced the psychosomatic and neurophysiological understanding [5,11,15].

#### 3.4.2. Epidemiology and Demographics

Population-based prevalence and incidence data are missing in the literature. All available epidemiological information is derived from clinic-based case series, practitioner surveys, or systematic reviews of case reports rather than from population studies [2,20]. Nonetheless, consistent demographic patterns emerged in the clinical cohorts.

In most case series, the mean age of patients was 50–60 years. Hara et al. (2012) reported a pooled mean age of approximately 51.7 years in the reviewed case reports [2]. Regarding sex distribution, a pronounced female predominance was consistently observed, and Hara et al. (2012) reported a female-to-male ratio of approximately 5.1:1 [2]. This pattern has been consistent across several subsequent reports [7,10,17].

Regarding clinical frequency, although the population prevalence remains unknown, OD appears to be an uncommon but clinically significant condition. A survey of US orthodontists found that approximately 50% were unfamiliar with the term “phantom bite.” However, many reported encountering patients with compatible complaints [20], suggesting under-recognition rather than true rarity.

OD was characterized by chronic persistence, with many patients reporting a symptom duration of several years, and Hara et al. (2012) reported a mean symptom duration > 6 years [2]. Symptoms began after dental procedures or occlusal interventions in approximately 75% of cases [16]; however, spontaneous onset was also reported [7,11].

#### 3.4.3. Clinical Presentation

The hallmark symptom is a persistent sensation that the bite is “wrong,” uncomfortable, or altered despite normal clinical and radiographic findings [1,2,6]. Patients typically reported a feeling that teeth do not fit together properly, awareness of specific teeth being “too high” or “in the way,” constant preoccupation with occlusal contacts, and inability to find a comfortable bite position. In addition, emotional distress, anxiety, and depression were frequently reported [2,11,12], and patients often exhibited obsessive checking behavior. Moreover, extensive consultation with multiple dental practitioners was common, with patients seeking repeated evaluations and treatments [3,6], and substantial impairments in oral health-related quality of life and general well-being were reported [12,13,17].

Although concurrent TMD has been reported, the signs and symptoms cannot fully explain the occlusal perception in many cases. TMD is typically characterized by pain and functional limitations rather than pure dysesthetic sensations [7,11,17]. Interestingly, several studies have reported high rates of psychiatric comorbidities, including major depressive, anxiety, somatoform, personality, and psychotic spectrum disorders [4,10,17]. Watanabe et al. (2015) specifically examined psychiatric comorbidities in PBS and reported heterogeneous psychiatric presentations and variable responses to psychopharmacological interventions [17].

Oguchi et al. (2017) reported outcomes in a Japanese cohort of 61 patients with OD managed with psychosomatic-oriented care [10]. Symptom resolution was achieved in 41% of patients, whereas 33% and 21% discontinued treatment (often with persistent complaints) and required referral to other specialties, respectively. Notably, patients who discontinued treatment often had more prominent psychiatric features.

#### 3.4.4. Etiology and Pathophysiology

The etiology of OD remains incompletely understood, with multiple, sometimes competing, mechanistic hypotheses. Early studies emphasized primary psychiatric etiologies, including monosymptomatic hypochondriasis, delusional disorders, and somatoform disorders [9,10]. Although psychiatric comorbidities are well-documented, contemporary studies generally reject purely psychiatric models in favor of more integrative frameworks [1,17].

Emerging evidence supports the primary role of altered central sensory processing. Imhoff et al. (2020) reported that OD is likely independent of the actual occlusion and probably reflects maladaptive central signal processing or occlusal hypervigilance [1]. Ono et al. (2016) used portable functional near-infrared spectroscopy (fNIRS) to study the prefrontal hemodynamic responses during occlusal interference tasks [18]. Patients with OD showed persistent increases in deoxygenated hemoglobin over the left frontal pole, and deoxygenated hemoglobin levels in channel-3 could discriminate patients with OD from controls with 92.9% accuracy. Furthermore, Umezaki et al. (2019) reported a case in which pharmacological treatment (mirtazapine plus aripiprazole) improved both symptoms and regional cerebral blood flow, supporting central nervous system involvement [23].

Munakata et al. (2016) reported that although occlusal recognition thresholds were similar between patients with OD and controls, discomfort thresholds were significantly lower in patients with OD, suggesting a heightened sensitivity to occlusal discomfort [19]. Some authors propose trigeminal neuropathy or altered peripheral sensory input following dental procedures [6], autoimmune or inflammatory processes affecting oral sensory pathways [6], and altered proprioceptive feedback from periodontal mechanoreceptors [14] as peripheral factors associated with OD. However, these peripheral hypotheses are speculative and lack robust empirical support.

The current consensus favors a multifactorial biopsychosocial model in which predisposing factors (personality traits and psychiatric vulnerability) interact with precipitating factors (dental procedures and life stressors) to trigger perpetuating factors (central sensitization, maladaptive coping, and iatrogenic interventions) that maintain the condition [1,10,17]

#### 3.4.5. Differential Diagnosis

An accurate diagnosis of OD requires the systematic exclusion of other conditions that may present with similar complaints, such as dental and occlusal conditions [1,6], including recent dental work with actual iatrogenic occlusal errors and painful dental conditions (pulpitis, periodontitis, or cracked tooth syndrome); temporomandibular disorders, such as myofascial pain and dysfunction, temporomandibular joint arthralgia or arthritis, and disc displacement disorders; neurological conditions, such as trigeminal neuralgia or neuropathic pain, burning mouth syndrome, atypical facial pain, or post-traumatic trigeminal neuropathy [6]; primary psychiatric disorders, including somatic symptom disorder, illness anxiety disorder, delusional disorder (somatic type), major depressive disorder with somatic focus, and obsessive–compulsive disorder [9,19]; other oral conditions, such as oral dyskinesia or movement disorders (xerostomia and salivary dysfunction); and altered oral sensation due to medication side effects. However, Imhoff et al. (2020) emphasized that the diagnosis of OD should be based on characteristic features rather than solely on exclusion [1].

#### 3.4.6. Diagnostic Approaches and Criteria

Several factors complicate the diagnosis of OD, including lack of validated, standardized diagnostic criteria; overlap with other conditions (TMD or psychiatric disorders); variable clinical presentations; limited clinician awareness and training [20]; and absence of definitive biomarkers or objective tests.

Although no universally accepted diagnostic criteria exist, based on a systematic review of case reports [2], Hara et al. (2012) proposed persistent uncomfortable or altered bite sensation, absence of objective occlusal discrepancy on clinical examination, symptom duration >6 months, onset following dental treatment, and presence of psychological distress or psychiatric comorbidity as unified diagnostic criteria.

Imhoff et al. (2020) provide similar diagnostic guidance, emphasizing the discrepancy between subjective complaints and objective findings as a cardinal feature [1]. The recommended assessment components include comprehensive history, including detailed symptom characterization, temporal pattern and onset circumstances, history of dental treatment and its effects, impact on daily functioning and quality of life, psychological and psychiatric history, and current medications; thorough clinical examination, including comprehensive oral and dental examination, systematic occlusal assessment (static and dynamic), temporomandibular joint and masticatory-muscle evaluation, neurological screening, and documentation of objective findings (or lack thereof); psychological screening, including assessment for depression, anxiety, and somatization; evaluation of illness beliefs and coping strategies; and consideration of formal psychiatric consultation.

In addition, emerging research has explored objective tests to aid diagnosis. A study on foil-thickness recognition and discomfort threshold testing reported that discomfort thresholds (but not recognition thresholds) were significantly lower in patients with OD [19]. Thus, the “foil grinding test” may provide a quantitative measure of occlusal perceptual sensitivity. Similarly, Ono et al. (2016) demonstrated that portable fNIRS measurement of prefrontal hemodynamic responses during occlusal interference could discriminate patients with OD from controls with high accuracy [18]. However, this technology is not yet clinically available, and requires validation using larger samples. Furthermore, research-level neuroimaging (cerebral blood flow assessment using functional magnetic resonance imaging) has provided insights into central nervous system involvement [23]. However, the technique is not practical for routine diagnosis.

#### 3.4.7. Treatment Approaches and Management

The literature consistently emphasizes conservative, multidisciplinary management, involving a general dentist or prosthodontist (for initial assessment and conservative management), psychologist or psychiatrist (for psychological assessment and treatment), orofacial pain specialist (for differential diagnosis and pain management), and physical therapist (in patients with TMD comorbidity). Several clinical guidelines and expert consensus statements provide recommendations for managing OD, though these are based on clinical experience rather than controlled trials (CEBM Level 5). Türp and Hellmann (2023) in their narrative review emphasized that the primary goal should be improving oral health-related quality of life rather than “curing” the occlusal sensation [15], and Kelleher et al. (2017) in their clinical review (case series) specifically warned about the “paradox” of patients demanding dental solutions to non-dental problems [4].

Imhoff et al. (2020) in their clinical guideline provided comprehensive management guidelines [1] comprising patient education, including explanation of the condition as a sensory perception disorder rather than a structural problem, reassurance regarding the absence of dental pathology, discussion of the biopsychosocial nature of symptoms, and setting realistic treatment expectations and avoiding irreversible dental interventions such as occlusal adjustments, extensive restorations, and orthodontic treatments [1,3,6,10] that often fail to provide relief and may worsen symptoms or create iatrogenic complications.

In addition, psychological and behavioral interventions are recommended. Among these, cognitive–behavioral therapy (CBT) is recommended as the primary treatment modality [1,6,10] for maladaptive illness beliefs and behaviors and teaches coping strategies and attention redirection. However, evidence is limited to case reports and expert consensus. In addition, counseling and psychoeducation are recommended for addressing distress, managing stress, and improving sleep hygiene and relaxation, and “defocusing” strategies can be used to redirect attention away from occlusal sensations. Although mindfulness-based approaches have been reported, evidence supporting their usefulness is limited.

Few case reports and small case series have reported the usefulness of pharmacological treatments, including antidepressants such as selective serotonin reuptake inhibitors, serotonin-norepinephrine reuptake inhibitors, and mirtazapine [23]; antipsychotics and mood stabilizers including low-dose atypical antipsychotics (e.g., aripiprazole) [23] for patients with psychotic features or severe somatization; and anxiolytics such as benzodiazepines (short-term use only, due to dependence risk) and buspirone or pregabalin (limited evidence).

Limited empirical evidence from observational studies provides support for specific interventions, though the quality of evidence remains low (CEBM Level 3–4). However, Watanabe et al. (2015) in their retrospective case series study reported heterogeneous responses to psychopharmacological interventions, emphasizing the need for individualized approaches [17].

A recurring theme in the literature is the risk of iatrogenic complications due to inappropriate dental interventions. Watanabe et al. (2021) described cases of “iatrogenic dental progression,” in which repeated dental procedures exacerbated PBS symptoms, particularly in patients with comorbid psychosis [3]. Moreover, several studies suggested that patients may become trapped in cycles of unsuccessful treatment, leading to extensive dental work, financial burdens, and worsening psychological distress [3,6,10], and some patients develop secondary dental pathology from excessive interventions. Although adjunctive dental treatments such as oral appliances/splints may provide temporary relief through “defocusing” effects [1,17], they should be used as supportive measures, and not as the primary treatment owing to the risk of reinforcing maladaptive illness beliefs. Reversible occlusal modifications such as temporary composite additions are generally not recommended [1]. However, they can be used diagnostically but not therapeutically.

Regarding treatment outcomes and prognosis, although individual case reports describe successful outcomes with various approaches, the heterogeneity of treatments and lack of controlled studies preclude definitive conclusions regarding efficacy. The largest cohort study by Oguchi et al. (2017) provides the most comprehensive outcome data backed by observational study [10]: 41%, 33%, and 21% of patients achieved resolution with psychosomatic management, discontinued treatment (often with persistent symptoms), and required referral or transfer to other specialties, respectively. Notably, patients with prominent psychiatric features had poorer outcomes. In addition, limited evidence suggests that longer symptom duration before appropriate diagnosis, history of multiple unsuccessful dental interventions, severe psychiatric comorbidities, rigid illness beliefs, and lack of insight into psychosomatic contributions may be associated with poorer outcomes. Conversely, early recognition, appropriate patient education, and timely multidisciplinary referral may improve prognosis [1,10].

Integrating evidence from both guidelines and observational studies, the following management approach is recommended:

- Strongly recommended (convergent evidence): Patient education about the condition’s nature, avoidance of irreversible occlusal interventions, psychiatric assessment and treatment of comorbidities, and a multidisciplinary care approach.
- Recommended with limited evidence: Cognitive behavioral therapy, pharmacotherapy (SSRIs, SNRIs, aripiprazole) in selected cases, and supportive psychotherapy.
- Strongly contraindicated (consistent evidence of harm): Repeated occlusal adjustments, extensive prosthodontic treatment, orthodontic treatment, and any irreversible dental interventions.

### 3.5. Summary of Evidence

Table 1 presents a comprehensive summary of all the included studies organized chronologically, including study characteristics, sample sizes, Oxford levels of evidence, key findings, and conclusions.

The evidence base for occlusal dysesthesia is primarily composed of low-quality evidence (CEBM Levels 4–5). There was no level 1 evidence found, which means there were no systematic reviews of randomized controlled trials. One study classified as Level 2 evidence (individual RCTs) was identified [2]. There is a scarcity of level 3 evidence derived from case–control studies and retrospective cohorts [18,19]. Most of the evidence (13 studies with Levels 4 and 5) comes from case series, case reports, and expert opinion [1,3,4,5,6,7,8,10,11,14,15,16,21,22,23].

Table 2 shows several consistent results from different studies, even though the study designs were different and the quality of the evidence was low.

## 4. Discussion

This scoping review advanced beyond previous narrative syntheses [1,11] by implementing systematic search methods across six databases, applying explicit eligibility criteria, using a structured study selection process, and conducting a formal evidence quality assessment according to the Oxford CEBM levels of evidence. Whereas narrative reviews provided valuable expert perspectives, the systematic approach adopted in this scoping review enhanced reproducibility, minimized selection bias, and yielded a more comprehensive mapping of the available evidence. To the authors’ knowledge, this paper represents the most current and comprehensive scoping review on occlusal dysesthesia, incorporating a systematic literature search completed through October 2025 and rigorous, prespecified evidence grading.

### 4.1. Comparison with Existing Literature

Hara et al. (2012) synthesized the findings in case reports and proposed unified diagnostic criteria [2]. Although our review confirmed their key findings, incorporating research data from the subsequent decade refined our understanding of the neurophysiological mechanisms and strengthened evidence for conservative management.

Melis and Zawawi (2015) emphasized the contraindication of irreversible treatments [6]. Our review supports this position with additional evidence from subsequent guidelines and cohort studies.

Imhoff et al. (2020) published the most comprehensive evidence-based clinical guidelines to date [1]. Our scoping review complements this guideline by systematically mapping the broader literature landscape and assessing the evidence quality using standardized criteria.

Tu et al. (2021, 2022) emphasized the psychosomatic nature of OD and multidisciplinary care needs [5,11]. Our review integrates these perspectives into a comprehensive evidence synthesis.

### 4.2. Clinical Implications

The findings in this review have several important clinical implications. General dentists and specialists should be aware of the characteristic presentation of OD to avoid diagnostic delays and inappropriate interventions. Key red flags include persistent occlusal complaints without objective findings, history of multiple unsuccessful dental treatments, psychological distress and preoccupation with occlusion, and repeated requests for occlusal adjustments.

Patients with OD often strongly believe that dental interventions will resolve their symptoms. Clinicians must resist this pressure and educate patients about the true nature of the condition [1,10]. In addition to dental examination, assessment should include psychological screening and consideration of psychosocial factors [11,17]. Moreover, dentists should develop relationships with psychologists, psychiatrists, and orofacial pain specialists to facilitate appropriate referrals [15]. Kelleher et al. (2017) discussed medicolegal risks for clinicians, noting that patients may pursue complaints or litigation when treatments fail [4]. Clear documentation, informed consent, and early appropriate referral are essential, particularly given the medicolegal risks associated with occlusal dysesthesia [4].

Mental health professionals, including psychologists and psychiatrists, should be aware that persistent occlusal complaints may represent specific somatic symptom presentations that require specialized management and that effective management requires close collaboration between mental health and dental professionals to address both somatic and psychological dimensions. In particular, CBT and other psychological treatments should be adapted to address the specific cognitive and behavioral patterns characteristic of OD.

Regarding healthcare systems and education, dental and medical curricula should include training on OD to improve recognition and appropriate management [20]. Most of the published literature originates from Japan, Europe, and North America. Therefore, cultural factors may influence symptom expression, help-seeking behavior, and treatment acceptability. Moreover, the structure of the healthcare system affects access to multidisciplinary care. Additionally, healthcare systems should establish clear diagnostic and referral pathways for patients with persistent somatic symptoms affecting the oral cavity. Where feasible, specialized clinics integrating dental, psychological, and medical expertise may optimize the care for complex cases.

### 4.3. Pathophysiological Insights and Future Directions

Recent neurophysiological studies provide intriguing insights into potential mechanisms that align with the broader pain neuroscience concepts of central sensitization and altered pain modulation. Our findings suggest that OD shares conceptual similarities with other conditions characterized by persistent somatic symptoms without a clear peripheral pathology, such as chronic pain conditions (fibromyalgia and irritable bowel syndrome), functional neurological disorders, persistent postural-perceptual dizziness, and tinnitus. Insights from these fields, particularly regarding central sensitization, predictive coding, and aberrant salience attribution, may inform future research on OD.

### 4.4. Research Gaps and Future Priorities

This scoping review identified substantial gaps in the current knowledge regarding OD. First, no population-based prevalence or incidence data are available. Therefore, large-scale epidemiological studies are required to determine the true prevalence in general and clinical populations, incidence rates and natural history, risk factors for development and persistence, and economic burden and healthcare utilization patterns. Second, prospective cohorts with extended follow-up are required to clarify the natural history and spontaneous remission rates, prognostic factors, and long-term outcomes of different management approaches. Third, larger, well-controlled neuroimaging studies using functional magnetic resonance imaging, positron emission tomography, or advanced fNIRS are required to validate and extend the preliminary findings, identify the specific neural circuits and neurotransmitter systems involved, and develop potential biomarkers for diagnosis or treatment response prediction. In addition, psychophysical studies with comprehensive sensory testing protocols are required to characterize the full spectrum of perceptual alterations, distinguish OD from related conditions, and provide quantitative outcome measures for treatment trials. Furthermore, investigation of potential genetic susceptibility factors and molecular mechanisms can inform personalized treatment approaches.

Regarding diagnostic research needs, the proposed diagnostic criteria [1,2] must be validated using prospective clinical studies involving diverse populations. Furthermore, development of standardized assessment tools, such as validated questionnaires and clinical assessment protocols, would facilitate consistent diagnosis across settings, severity quantification, and treatment outcome measurement. Similarly, further development and validation of objective tests (fNIRS and sensory thresholds) could improve diagnostic accuracy, reduce diagnostic delay, and provide mechanistic insights.

To assess treatment efficacy, high-quality randomized controlled trials focusing on psychological interventions (CBT, acceptance and commitment therapy, and mindfulness), pharmacological treatments (antidepressants, antipsychotics, and neuromodulators), combined approaches, and novel interventions based on mechanistic understanding are urgently required. Particularly, studies comparing different management strategies would inform optimal treatment algorithms, and studies elucidating effective treatments can guide the development of more targeted interventions. Finally, studies evaluating whether early intervention after dental procedures can prevent OD development in at-risk individuals are warranted.

Regarding health services research needs, studies on effective implementation of evidence-based management in real-world clinical settings are required. In addition, studies evaluating the efficacy of educational interventions for improving clinicians’ recognition and management of OD are required. Finally, studies evaluating the cost-effectiveness of different management approaches are required to inform healthcare policies and resource allocation.

### 4.5. Geographical and Cultural Considerations

A significant finding of this scoping review is the unequal contribution of Japanese literature to the evidence base regarding occlusal dysesthesia. Despite our thorough search of international databases, ten (50%) of the 20 studies we looked at came from Japan [2,3,5,7,10,11,17,18,19,23]. This geographic concentration prompts significant inquiries regarding the impact of cultural factors on the recognition, reporting, or clinical presentation of OD. The Japanese healthcare system offers universal coverage with minimal barriers to dental care, enabling patients to obtain multiple opinions, which may result in heightened detection rates of intricate conditions such as OD [10]. Furthermore, the Japanese dental education system has historically emphasized thorough occlusal analysis, which may increase clinicians’ awareness of patients’ occlusal issues [18].

In Japan, there are dedicated research groups that are interested in OD, especially at Tokyo Medical and Dental University which has led to significant number of publications [3,5,11,17]. This research tradition may indicate both clinical necessity and scholarly interest in psychosomatic dentistry, a discipline with significant representation in Japanese dental education. The geographical concentration of published studies indicates a need for broader international research. Clinicians and researchers globally should be aware of this condition and evaluate whether analogous patients within their populations may be inadequately recognized or diagnosed using alternative terminology. The establishment of international consensus regarding diagnostic criteria and treatment methodologies necessitates contributions from various cultural and healthcare contexts.

### 4.6. Strengths and Limitations

#### 4.6.1. Strengths

This study adopted a comprehensive search strategy in which multiple databases were searched using systematic methods according to the PRISMA guidelines (2020). Moreover, the scoping review approach allowed the inclusion of diverse study types and provided a comprehensive overview of the literature, and the systematic application of Oxford CEBM levels of evidence provided a transparent evaluation of evidence quality. Furthermore, the incorporation of the recent German guidelines [1] ensured the inclusion of the most current expert consensus. Finally, organizing evidence chronologically illuminated the evolution of understanding over time.

#### 4.6.2. Limitations

Although studies published in the English and German languages were included, relevant literature in other languages (particularly Japanese, given the substantial Japanese contributions to this field) may have been missed. Moreover, grey literature, such as conference abstracts and unpublished studies, was not systematically searched, potentially introducing publication bias. In addition, historical variations in terminology may have resulted in some relevant studies being missed despite comprehensive search terms. This may have led to inadvertent exclusion of relevant studies using non-standard terminology, despite our comprehensive search strategy employing multiple term variants. Furthermore, substantial heterogeneity in the study design, population, and outcome measures precluded a quantitative meta-analysis, and the predominance of low-level evidence (case reports and series) limited the confidence in many findings and limits the strength of treatment recommendations. Additionally, the use of different methods in different studies made it impossible to standardize data extraction and quantitative synthesis. However, this lack of standardization is itself an important finding that shows how important it is to have standardized diagnostic criteria and assessment protocols.

## 5. Conclusions

OD is a complex biopsychosocial condition characterized by persistent uncomfortable bite sensations without an objective occlusal pathology. This scoping review revealed that, although clinical recognition has improved and conceptual understanding has evolved toward neurophysiological and biopsychosocial models, the majority evidence is low-level.

The key conclusions are as follows:

1. OD is a distinct clinical entity with the following characteristic features: female predominance, onset in middle age, chronic course, onset often following dental procedures, and frequent psychiatric comorbidities.
2. Current evidence suggests that OD is a central sensory processing disorder rather than a primary occlusal or purely psychiatric condition, although psychiatric comorbidities are common and clinically important.
3. Conservative multidisciplinary management is strongly recommended, including patient education, CBT, and supportive pharmacotherapy. Irreversible dental interventions should be avoided because of the risk of iatrogenic harm.
4. Emerging neurophysiological evidence (fNIRS and cerebral blood flow studies) provides objective support for central nervous system involvement and may eventually yield diagnostic biomarkers.
5. Substantial research gaps exist, particularly the absence of population-based epidemiological data, validated diagnostic criteria, and randomized controlled trials.
6. Improved professional education and clinical awareness are needed to facilitate the early recognition, appropriate management, and prevention of iatrogenic complications.

This scoping review provides the most up-to-date synthesis of evidence on occlusal dysesthesia, with a systematic search through October 2025, systematically mapping the current state of knowledge and identifying critical gaps that should guide future research priorities. Future research should include population-based epidemiological studies, mechanistic neuroimaging investigations, studies focusing on the development and validation of diagnostic criteria and assessment tools, and high-quality randomized controlled trials focusing on psychological and pharmacological interventions. Such research is essential to establish evidence-based standards of care for this challenging condition.

For clinicians encountering patients with persistent uncomfortable bite sensations, the key message is clear: recognize the condition early, resist pressure for dental solutions for what is fundamentally a disorder of sensory perception and processing, educate patients about the true nature of their symptoms, and facilitate timely multidisciplinary care to optimize outcomes and prevent iatrogenic harm.

## Figures and Tables

**Table 1:** Characteristics of the included studies.

Author(s), Year	Title	Type of Study	Sample Size	Oxford CEBM Level	Important Findings	Conclusion
Yamaguchi et al., 2007 [7]	A clinical study on persistent uncomfortable occlusion	Retrospective case series	39 patients	Level 4	Improvement observed in 17/39 patients; muscle relaxant medication showed significant relationship with treatment outcome; prognosis cannot be predicted from initial presentation alone	Persistent uncomfortable occlusion comprises several occlusal patterns; comprehensive assessment needed beyond occlusal contacts
Ligas et al., 2011 [20]	Phantom bite: A survey of US orthodontists	Cross-sectional survey	337 orthodontists	Level 4		Awareness of phantom bite among orthodontists is inconsistent; educational efforts needed
Hara et al., 2012 [2]	Occlusal dysesthesia: A qualitative systematic review of the epidemiology, aetiology and management	Systematic review	37 patients (pooled)	Level 2	Predominantly middle-aged women; long symptom duration (mean, ~6 years); frequent psychological comorbidity; treatments (psychotherapy, CBT, splints, medications) supported mainly by case reports	Current evidence is low quality; OD has characteristic features, but robust studies needed for mechanisms and evidence-based management
Leon-Salazar et al., 2012 [8]	Pain and persistent occlusal awareness: What should dentists do?	Clinical discussion (Case report)	1 patient	Level 5	Cases combining TMD and phantom bite syndrome; multidisciplinary diagnostic context important	Dentists should recognize persistent occlusal awareness and consider multidisciplinary evaluation rather than repeated occlusal treatments
Melis et al., 2015 [6]	Occlusal dysesthesia: a topical narrative review	Topical narrative review	22 articles reviewed	Level 5	OD commonly associated with emotional distress; frequently follows dental procedures; repeated dental interventions often worsen symptoms	Irreversible occlusal treatments contraindicated; management should emphasize counseling, defocusing, psychotherapy, conservative measures
Watanabe et al., 2015 [17]	Psychiatric comorbidities and psychopharmacological outcomes of phantom bite syndrome		130 patients	Level 4	High frequency of psychiatric comorbidities; variable responses to psychopharmacological interventions	Psychiatric evaluation is important in PBS management; psychopharmacology may benefit some patients but responses heterogeneous
Tinastepe et al., 2015 [21]	Phantom bite: a case report and literature review	Case report	1 patient	Level 5	Patient with persistent bite discomfort without objective dental pathology; sertraline 50 mg/day + psychotherapy led to significant improvement	Clinicians should be aware of phantom bite to avoid unnecessary interventions; consider non-dental causes
Ono et al., 2016 [18]	Diagnosis of occlusal dysesthesia utilizing prefrontal hemodynamic activity with slight occlusal interference	Case–control (fNIRS study)	6 OD patients, 8 controls	Level 3	OD patients showed persistent increases in deoxyhemoglobin over left frontal pole during occlusal loading; 92.9% diagnostic accuracy	Prefrontal hemodynamic responses may provide objective neuronal signature for OD diagnosis
Munakata et al., 2016 [19]	Relationship between occlusal discomfort syndrome and occlusal threshold	Case–control study	21 ODS patients, 21 controls	Level 3	Recognition thresholds similar between groups; discomfort thresholds significantly lower in ODS patients	Occlusal discomfort threshold (not recognition) is altered in ODS; foil grinding test useful clinical tool
Kelleher et al., 2017 [4]	The paradoxes of phantom bite syndrome or occlusal dysaesthesia	Clinical review (Case series)	12 patients	Level 5	PBS typically driven by fixed belief about bad occlusion; often leads to multiple unsuccessful dental interventions; psychiatric explanations discussed	Early recognition and referral to secondary care (including psychiatry) recommended to prevent unnecessary procedures
Oguchi et al., 2017 [10]	Occlusal dysesthesia: A clinical report on psychosomatic management of a Japanese patient cohort	Retrospective cohort study	61 patients	Level 4	Among 61 patients, 41% reported resolution, 33% discontinued treatment, 21% were referred to specialists, 5% continued treatment; mean follow-up 3.6 years	
Sutter, 2017 [22]	Phantom bite: a real or a phantom diagnosis? A case report	Case report	1 patient	Level 5	Digital occlusal analysis identified prolonged disclusion time and force imbalance; targeted corrections produced marked symptom improvement	Some phantom bite presentations may have identifiable occlusal factors amenable to careful, guided correction
Umezaki et al., 2019 [23]	Change of cerebral blood flow after successful pharmacological treatment of phantom bite syndrome: A case report	Case report (with SPECT imaging)	1 patient	Level 5	Aripiprazole 3 mg/day led to symptomatic improvement and normalized regional cerebral blood flow on SPECT	Findings support CNS involvement in PBS; psychopharmacological treatment may alter neurophysiologic correlates
Imhoff et al., 2020 [1]	Occlusal dysesthesia—A clinical guideline	Clinical guideline	77 articles reviewed	Level 5	OD independent of occlusion; likely due to maladaptive signal processing; recommended non-dental therapies (education, CBT, supportive medication); advised against irreversible dental treatments	Treat OD with patient education, defocusing, CBT, supportive pharmacotherapy; avoid irreversible dental interventions
Tu et al., 2021 [11]	Phantom bite syndrome: Revelation from clinically focused review	Narrative review	Not applicable	Level 5	PBS is rare but has consistent clinical pattern: persistent non-verifiable occlusal complaints, frequent psychiatric comorbidity, substantial QoL impact	Increased clinician awareness and multidisciplinary care model (dentists, psychiatrists, psychotherapists) necessary
Watanabe et al., 2021 [3]	Case Report: Iatrogenic Dental Progress of Phantom Bite Syndrome: Rare Cases With the Comorbidity of Psychosis	Case series	3 patients	Level 5	Iatrogenic dental interventions sometimes precipitated or worsened PBS symptoms in patients with comorbid psychosis	Dental procedures can exacerbate PBS in vulnerable patients; coordination with psychiatric services critical
Tu et al., 2022 [5]	Phantom bite syndrome	Clinical review	Not applicable	Level 5	Summarizes clinical features of PBS; emphasizes psychosomatic impact and risk of unnecessary dental interventions	Awareness and conservative multidisciplinary management are essential to limit harm from unnecessary treatments
Türp et al., 2023 [15]	Occlusal dysesthesia and its impact on daily practice	Narrative review	Not applicable	Level 5	OD commonly occurs after dental therapy in patients with stress; often with TMD comorbidity; occlusal adjustments contraindicated; psychological treatments recommended	Primary goal is to improve oral health-related QoL with counseling, psychological therapy, splint defocusing, medication rather than occlusal modification
Rampello et al., 2025 [14]	Description of low-threshold mechanisms of consciousness and occlusal dysesthesia: diagnosis and therapy through active functional rehabilitative repositioning bite	Theoretical article	Not applicable	Level 5	Proposes neurophysiological regulatory pathways; emphasizes occlusal hypervigilance as mechanism in OD; suggests active rehabilitative mandibular repositioning bites	Active rehabilitative mandibular repositioning bites may address occlusal hypervigilance, but empirical validation needed
Versteegh et al., 2025 [16]	Occlusal dysaesthesia: an unusual, persistent somatic symptom	Clinical guideline	Not applicable	Level 5	Approximately 75% of cases occurred after dental treatment; persistent symptoms should be treated as persistent somatic symptoms requiring dialogue and referral; routine occlusal adjustments contraindicated	General practitioners should engage patients and refer for interventions optimizing conditions for symptom resolution

CEBM, Oxford Centre for Evidence-Based Medicine; US, United States of America; CBT, cognitive–behavioral therapy; OD, Occlusal dysesthesia; QoL, Quality of Life; PBS, Phantom bite syndrome; TMD, Temporomandibular Disorders.

**Table 2:** Summary of Key Findings.

**Domain**	**Key Findings**	**Evidence Quality (CEBM Level)**	**Clinical Implications**	**Research Gaps**
Epidemiology and Demographics	Predominantly affects middle-aged women (60–80% female) Typical age of onset: 40–60 years Long symptom duration (often >2 years) Prevalence in general population unknown	Level 4–5 (Case series, surveys, expert opinion)	Clinicians should maintain high index of suspicion in middle-aged female patients with persistent occlusal complaints Condition is likely under-recognized	Large-scale epidemiological studies needed Prevalence data in general population Risk factor identification studies
Etiology and Pathophysiology	Central sensory processing abnormality (not primary occlusal problem) High rates of psychiatric comorbidity (depression 40–60%, anxiety disorders common) Possible prefrontal cortex dysfunction Maladaptive neuroplastic changes	Level 3–5 (Case–control studies, case series, neuroimaging case reports)	Treatment should target central mechanisms, not peripheral occlusion Psychiatric screening essential Multidisciplinary approach required	Neuroimaging studies with larger samples Prospective cohort studies Biomarker identification Pathophysiological mechanism clarification
Clinical Presentation	Persistent sensation of “wrong” or uncomfortable bite Absence of objective occlusal discrepancies Often triggered by dental treatment Symptoms worsen with dental interventions Significant impact on quality of life	Level 4–5 (Case series, case reports)	Detailed history essential (onset after dental treatment is red flag) Objective occlusal examination typically normal Avoid repeated occlusal adjustments	Standardized symptom assessment tools Natural history studies Quality of life impact quantification
Differential Diagnosis	Must distinguish from: - True occlusal abnormalities - TMD - Burning mouth syndrome - Other orofacial pain conditions - Primary psychiatric disorders	Level 5 (Expert opinion, clinical guidelines)	Comprehensive differential diagnosis is essential May coexist with other conditions Careful clinical examination required	Diagnostic algorithm development Inter-rater reliability studies Validation of diagnostic criteria
Diagnostic Approaches	No validated diagnostic criteria exist Diagnosis primarily by exclusion Psychiatric assessment recommended Objective occlusal analysis shows no abnormalities Prefrontal hemodynamic activity may show differences (experimental)	Level 4–5 (Case–control studies, expert opinion)	Diagnosis is clinical, based on history and exclusion of organic pathology Psychiatric consultation valuable Avoid over-reliance on occlusal analysis technology	Validated diagnostic criteria urgently needed Standardized assessment protocols Biomarker research Diagnostic test accuracy studies
Treatment and Management	Conservative approaches: Patient education and reassurance Cognitive behavioral therapy (CBT) Pharmacotherapy: SSRIs, SNRIs, atypical antipsychotics (aripiprazole most studied) Supportive psychotherapy Strong recommendation AGAINST: Irreversible occlusal adjustments Extensive prosthodontic treatment Orthodontic treatment Repeated dental interventions	Level 3–5 (Retrospective cohort, case series, expert opinion) Note: No RCTs exist	Critical: Avoid irreversible dental interventions (often worsen symptoms) Multidisciplinary care essential (dentist, psychiatrist, psychologist) Set realistic expectations (symptom management vs. cure) Pharmacotherapy may be beneficial in selected cases	Randomized controlled trials urgently needed Treatment outcome studies Comparative effectiveness research Long-term follow-up studies Optimal pharmacotherapy regimens
Prognosis and Complications	Chronic, often refractory condition Iatrogenic worsening common with inappropriate treatment Significant psychological distress Some patients respond to conservative management Spontaneous resolution rare	Level 4–5 (Case series, expert opinion)	Early recognition and appropriate management crucial to prevent iatrogenic harm Long-term supportive care often required Multidisciplinary approach improves outcomes	Prognostic factor identification Long-term outcome studies Predictors of treatment response Prevention of iatrogenic complications
Special Populations	Higher prevalence in Japan (possible cultural/diagnostic factors) Limited data on other populations No pediatric cases reported Male patients may be under-recognized	Level 5 (Expert opinion, case reports)	Cultural competence important in diagnosis Consider condition in diverse populations Male patients may present differently	Cross-cultural studies Studies in diverse populations Investigation of gender differences Pediatric/adolescent data

CEBM, Oxford Centre for Evidence-Based Medicine; RCT, Randomized Controlled Trial; TMD, Temporomandibular Disorder; CBT, Cognitive Behavioral Therapy; SSRI, Selective Serotonin Reuptake Inhibitor; SNRI, Serotonin–Norepinephrine Reuptake Inhibitor.

## Data Availability

The original contributions presented in this study are included in the article. Further inquiries can be directed to the corresponding author.

## Supplementary Materials

The following supporting information can be downloaded at: https://www.mdpi.com/article/10.3390/dj14010047/s1. File S1:Scoping Reviews (PRISMA-ScR) Checklist.

## Abbreviations

The following abbreviations are used in this manuscript:

OD	Occlusal dysesthesia
PBS	Phantom Bite Syndrome
CEBM	Center for Evidence-Based Medicine
TMD	Temporomandibular Disorders
fNIRS	Functional Near-Infrared Spectroscopy
CBT	Cognitive–Behavioral Therapy

## References

[1] Imhoff B., Ahlers M.O., Hugger A., Lange M., Schmitter M., Ottl P., Wolowski A., Türp J.C. (2020). Occlusal dysesthesia-A clinical guideline. J. Oral Rehabil..

[2] Hara E.S., Matsuka Y., Minakuchi H., Clark G.T., Kuboki T. (2012). Occlusal dysesthesia: A qualitative systematic review of the epidemiology, aetiology and management. J. Oral Rehabil..

[3] Watanabe M., Hong C., Liu Z., Takao C., Suga T., Tu T.T.H., Yoshikawa T., Takenoshita M., Sato Y., Higashihori N. (2021). Case Report: Iatrogenic Dental Progress of Phantom Bite Syndrome: Rare Cases with the Comorbidity of Psychosis. Front. Psychiatry.

[4] Kelleher M.G., Rasaratnam L., Djemal S. (2017). The paradoxes of phantom bite syndrome or occlusal dysaesthesia (‘dysesthesia’). Dent. Update.

[5] Tu T.T.H., Watanabe M., Toyofuku A., Yojiro U. (2022). Phantom bite syndrome. Br. Dent. J..

[6] Melis M., Zawawi K.H. (2015). Occlusal dysesthesia: A topical narrative review. J. Oral Rehabil..

[7] Yamaguchi T., Mikami S., Okada K., Matsuki T., Gotouda A., Gotuda S., Satoh K., Komatsu K. (2007). A clinical study on persistent uncomfortable occlusion. Prosthodont. Res. Pract..

[8] Leon-Salazar V., Morrow L., Schiffman E.L. (2012). Pain and persistent occlusal awareness: What should dentists do?. J. Am. Dent. Assoc..

[9] Kelleher M.G.D., Canavan D. (2020). The perils of “phantom bite syndrome” or “occlusal dysaesthesia”. J. Ir. Dent. Assoc..

[10] Oguchi H., Yamauchi Y., Karube Y., Suzuki N., Tamaki K. (2017). Occlusal dysesthesia: A clinical report on the psychosomatic management of a Japanese patient cohort. Int. J. Prosthodont..

[11] Tu T.T.H., Watanabe M., Nayanar G.K., Umezaki Y., Motomura H., Sato Y., Toyofuku A. (2021). Phantom bite syndrome: Revelation from clinically focused review. World J. Psychiatry.

[12] Tricco A.C., Lillie E., Zarin W., O’Brien K.K., Colquhoun H., Levac D., Moher D., Peters M.D.J., Horsley T., Weeks L. (2018). PRISMA Extension for Scoping Reviews (PRISMA-ScR): Checklist and explanation. Ann. Intern. Med..

[13] Howick J., Chalmers I., Glasziou P., Greenhalgh T., Heneghan C., Liberati A., Moschetti I., Phillips B., Thornton H. The 2011 Oxford CEBM Levels of Evidence (Introductory Document). Oxford Centre for Evidence-Based Medicine. https://www.cebm.ox.ac.uk/resources/levels-of-evidence/ocebm-levels-of-evidence.

[14] Rampello A., Rampello A. (2025). Description of low-threshold mechanisms of consciousness and occlusal dysesthesia: Diagnosis and therapy through active functional rehabilitative repositioning bite. Ann. Stomatol..

[15] Türp J.C., Hellmann D. (2024). Occlusal dysesthesia and its impact on daily practice. Semin. Orthod..

[16] Versteegh P.A.M., van Rood Y.R. (2025). Occlusale dysesthesie: Een bijzondere aanhoudende lichamelijke klacht [Occlusal dysaesthesia an unusual persistent somatic symptom]. Ned. Tijdschr. Tandheelkd..

[17] Watanabe M., Umezaki Y., Suzuki S., Miura A., Shinohara Y., Yoshikawa T., Sakuma T., Shitano C., Katagiri A., Sato Y. (2015). Psychiatric comorbidities and psychopharmacological outcomes of phantom bite syndrome. J. Psychosom. Res..

[18] Ono Y., Ishikawa Y., Munakata M., Shibuya T., Shimada A., Miyachi H., Wake H., Tamaki K. (2016). Diagnosis of occlusal dysesthesia utilizing prefrontal hemodynamic activity with slight occlusal interference. Clin. Exp. Dent. Res..

[19] Munakata M., Ono Y., Hayama R., Kataoka K., Ikuta R., Tamaki K. (2016). Relationship between occlusal discomfort syndrome and occlusal threshold. Kokubyo Gakkai Zasshi.

[20] Ligas B.B., Galang M.T.S., BeGole E.A., Evans C.A., Klasser G.D., Greene C.S. (2011). Phantom bite: A survey of US orthodontists. Orthodontics.

[21] Tinastepe N., Küçük B.B., Oral K. (2015). Phantom bite: Case report and literature review. Cranio.

[22] Sutter B.A. (2017). Phantom bite: A real or a phantom diagnosis? A case report. Gen. Dent..

[23] Umezaki Y., Tu T.T.H., Toriihara A., Sato Y., Naito T., Toyofuku A. (2019). Change of cerebral blood flow after a successful pharmacological treatment of phantom bite syndrome: A case report. Clin. Neuropharmacol..

